# Preventive effect of transcutaneous electrical acupoint stimulation pretreatment on lymphedema in patients undergoing axillary lymph node dissection for breast cancer: a randomized controlled trial protocol

**DOI:** 10.3389/fonc.2026.1816750

**Published:** 2026-04-29

**Authors:** Linna Wu, Qing Mao, Zhu Zhu, Jun Yang, Liang Chen, Xingtai Hu, Qilian Xia, Yongliang Wang, Shiyi Tang, Yu Zhou, Aimei Jiang, Rong Zhao

**Affiliations:** 1 The First Clinical Medical School, Yunnan University of Chinese Medicine, Kunming, Yunnan, China; 2 The First Affiliated Hospital, Kunming Medical University, Kunming, China; 3 Xuanwei Hospital of Traditional Chinese Medicine, Xuanwei, China; 4 Yunnan Provincial Hospital of Traditional Chinese Medicine, Kunming, Yunnan, China; 5 Department of Basic Medical Sciences, Shanghai Medical College, Fudan University, Shanghai, China; 6 The Second Clinical Medical School, Yunnan University of Chinese Medicine, Kunming, Yunnan, China

**Keywords:** axillary lymph node dissection for breast cancer, clinical randomized controlled trial, lymphedema, pretreatment, transcutaneous electrical acupoint stimulation

## Abstract

**Introduction:**

Breast cancer-related lymphedema (BCRL) is a serious and intractable complication that severely affects the quality of life and survival rates of cancer patients. Transcutaneous electrical acupoint stimulation (TEAS) has been applied in China for the treatment of lymphedema. However, there is currently no high-quality evidence-based data confirming the efficacy, safety, and preventive therapeutic role of TEAS for BCRL. This study aims to evaluate the efficacy and safety of TEAS pretreatment for BCRL through a multicenter, prospective, randomized, sham-controlled, parallel-group clinical trial, thereby providing evidence-based medical evidence.

**Methods and analysis:**

A total of 72 breast cancer participants scheduled for axillary lymph node dissection will be recruited from Yunnan Provincial Hospital of Traditional Chinese Medicine and the First Affiliated Hospital of Kunming Medical University. They will randomly assigned in a 1:1 ratio to the TEAS group or the Sham TEAS group, with 36 cases in each group. Treatment will be administered once daily from the first day before surgery to the third day after surgery (paused on the surgery day), and subsequently twice a week for 4 consecutive weeks, totaling 12 intervention sessions. This will be followed by an 18-month follow-up period. The primary outcome measures are the incidence and time of onset of lymphedema within 18-month postoperatively. Secondary outcome measures include: VAS scores for perceived swelling and lymphedema-related symptoms evaluated at baseline (T0), from preoperative to postoperative day 3 (T1-T4), upon completion of twice-weekly post-discharge treatments (T5-T12), and during monthly follow-ups until 18 months postoperatively (T13-T29); changes in circumference, edema stage, and severity of the affected limb evaluated at T0, before and after treatments from preoperative to postoperative day 3 (T1a/b-T4a/b), before and after twice-weekly post-discharge treatments (T5a/b-T12a/b), and at T29; skin thickness ultrasound and lymphangiography of the affected limb performed at T0, T2, T12, and T29; and serum inflammatory factor levels measured at T0, T4, and T12. Adverse events occurring during the trial will be recorded.

**Discussion:**

This study aims to evaluate the efficacy and safety of TEAS pretreatment for BCRL. If effective, TEAS will become a non-invasive adjunct therapy for preventing BCRL.

**Clinical trial registration:**

Chinese Clinical Trial Registry [website], identifier ChiCTR2400088117.

## Introduction

Breast cancer-related lymphedema (BCRL) is one of the relatively common complications following breast cancer surgery and falls under the category of “Edema” (Shuizhong) in Traditional Chinese Medicine (TCM). Its incidence rate can reach 6% to 63% (1, 2). Di Sipio et al. found that the probability of developing lymphedema in patients undergoing axillary lymph node dissection (ALND) is five times higher than in those undergoing Sentinel Lymph Node Biopsy (3). The incidence of BCRL following ALND is as high as 58.3% (4). The onset of lymphedema typically occurs between 3 months and 3 years postoperatively, peaking at 18 months. The incidence rate can reach 77% by 3 years postoperatively, followed by a small annual increase thereafter (5). Breast cancer patients who undergo ALND, sentinel lymph node biopsy, axillary radiation, and systemic chemotherapy face a lifelong risk of developing BCRL (6). Lymphedema is a chronic process; any factor causing obstruction or interruption of lymphatic drainage contributes to its occurrence (7). Since the treatment of breast cancer patients in China is predominantly based on comprehensive therapy involving modified radical mastectomy, chemotherapy, and radiotherapy, the incidence and severity of postoperative lymphedema are further exacerbated (8).

Currently, Western medical preventive measures mainly include preventive intermittent pneumatic compression (IPC) and preventive manual lymphatic drainage (MLD), limb rehabilitation exercises, and precautionary measures. These precautions include avoiding blood pressure measurement, blood draws, and injections on the affected upper limb; preventing burns, cuts, infections, and insect bites on the affected limb; avoiding saunas or hot baths; avoiding strenuous repetitive movements with resistance; and avoiding excessive fatigue or maintaining a fixed posture for prolonged periods (9). Preventive measures for BCRL are currently singular, and TCM involvement is primarily concentrated on treatment, with few reports on prevention. If patients miss the optimal window for conservative treatment and physiological lymphatic reconstruction, the condition may progress to a stage characterized by tissue proliferation, fat deposition, tissue fibrosis, and even ulceration or leakage, leaving surgery as the only option. However, current surgical interventions for BCRL involve high technical difficulty and long duration, and there is currently no ideal surgical procedure applicable to all patients with varying degrees of severity (10). Shifting the focus of BCRL management forward to the prevention stage allows patients to derive the maximum benefit. Therefore, actively exploring effective TCM preventive means to reduce the incidence of BCRL will not only break the limitation of singular preventive measures but also greatly benefit patients’ quality of life and promote the integrated development of Traditional Chinese and Western Medicine.

TCM has achieved significant clinical efficacy through its holistic concept and syndrome differentiation-based treatment (Bian Zheng Lun Zhi). It possesses unique advantages in treating edema; as early as the Inner Canon of the Yellow Emperor (Nei Jing), it was proposed that removing blood stasis through acupuncture and bloodletting could treat edema (11). Transcutaneous electrical acupoint stimulation (TEAS) is based on acupuncture but replaces needles with electrode pads, forming a current loop between two pairs of electrodes to stimulate acupoints, achieving effects identical to needle insertion (12). This therapy features controllable parameters, ease of standardized operation, and good reproducibility, and it can significantly save manpower costs associated with manual acupuncture manipulation. It allows for accurate regulation of stimulation intensity and can replace manual needle manipulation to save labor. As a novel therapy combining traditional acupuncture with modern electronic technology, TEAS has the advantages of being simple and non-invasive. Compared to manual acupuncture and electroacupuncture, TEAS does not require direct skin penetration, avoiding the risks of pain and infection, making it more easily accepted by patients. As a non-invasive treatment, it is also more readily accepted by surgical patients, and medical staff with basic training can operate it easily (13, 14). The physiological effects of TEAS are similar to those of electroacupuncture and manual acupuncture, capable of regulating the autonomic nervous system (15). Research by Jiang et al. indicated that electroacupuncture, TEAS, and manual acupuncture can all increase brain network connectivity, with TEAS showing superior effects (16). TEAS is widely applied clinically as a new acupuncture technology. Modern research also demonstrates that TEAS combined with other therapies has achieved significant efficacy in treating BCRL (17). Therefore, this study is designed as a clinical randomized controlled trial (RCT) to observe the preventive effect of TEAS pretreatment on patients undergoing ALND for breast cancer, aiming to provide new ideas and methods for the clinical prevention of BCRL.

## Methods and analysis

### Study design

**Figure 1:**
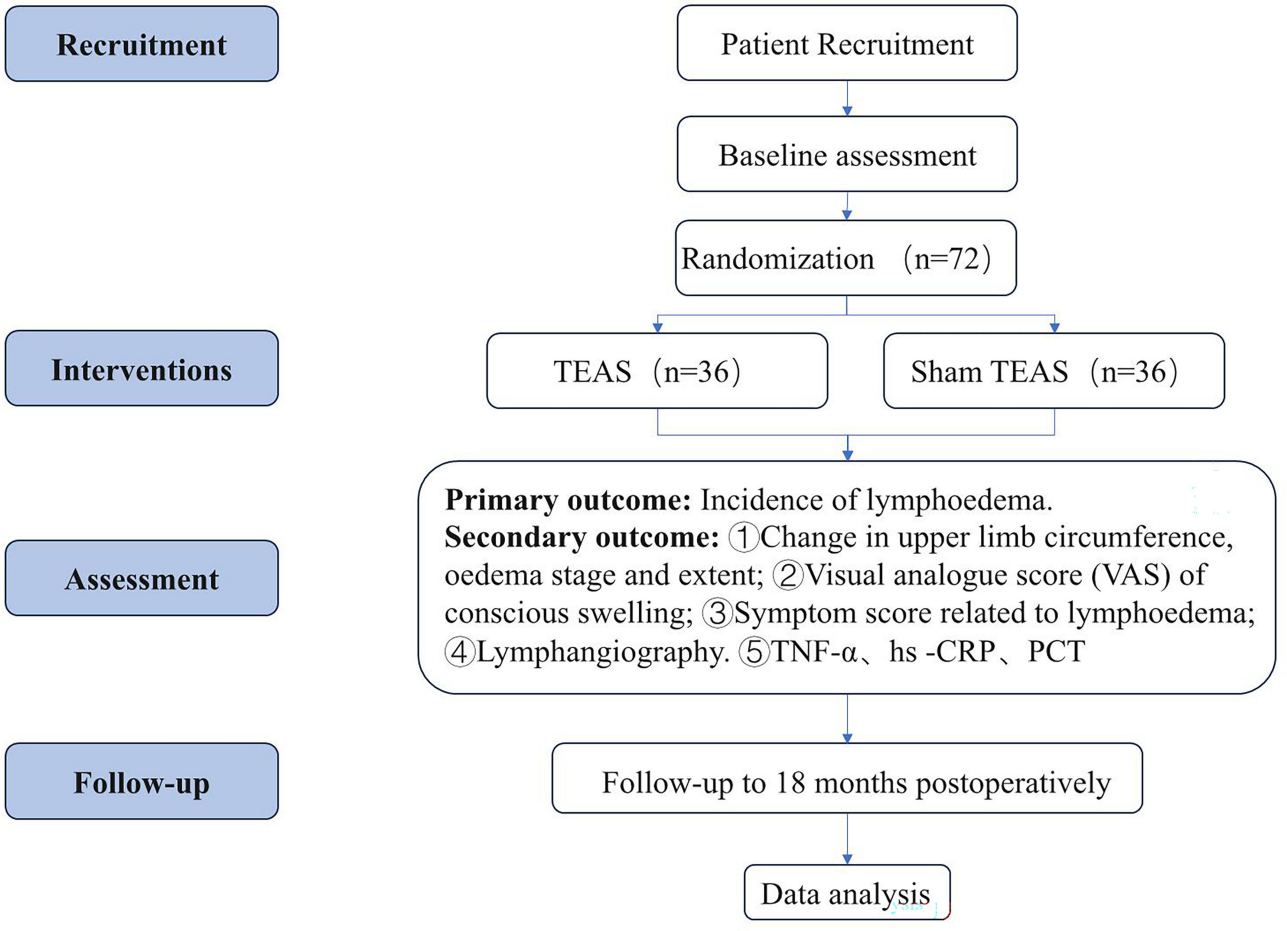
Trial flow diagram. TEAS, transcutaneous electrical acupoint stimulation; TNF-α, tumor necrosis factor-α; hs-CPR, high-sensitivity C-reactive protein; PCT, procalcitonin.

**Figure 2:**
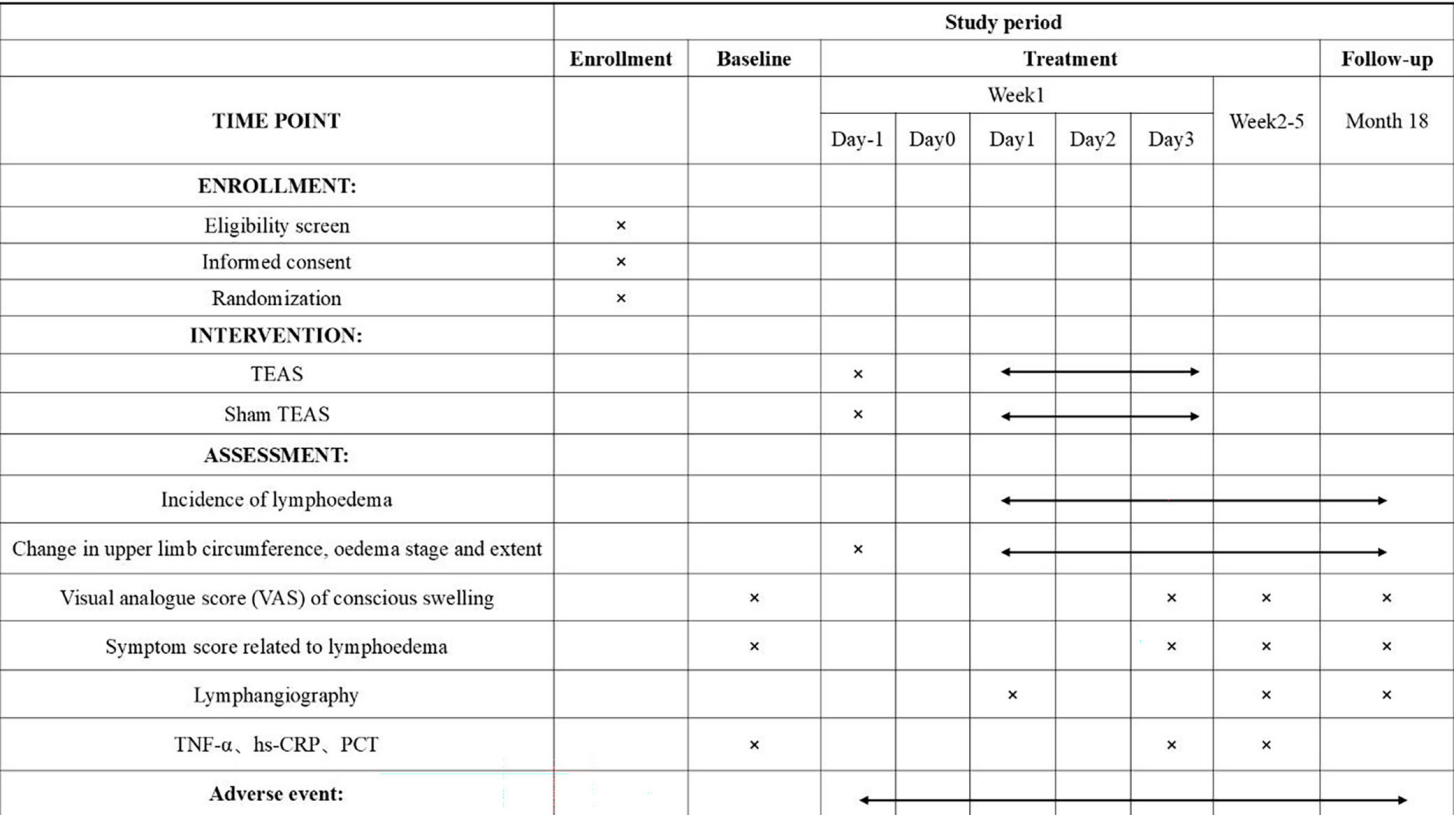
Schedule of enrolment, interventions and assessments.

This study is a multicenter, prospective, randomized, sham-controlled, parallel-group clinical trial designed to evaluate the efficacy and safety of TEAS pretreatment for lymphedema in patients undergoing ALND for breast cancer. A total of 72 eligible participants will be recruited and randomly assigned in a 1:1 ratio to either the TEAS group or the Sham TEAS group. The study protocol adheres to the Consolidated Standards of Reporting Trials (CONSORT) 2025 statement and the Standards for Reporting Interventions in Clinical Trials of Acupuncture (STRICTA) guidelines. The trial flow is illustrated in **Figure 1**, and the schedule for participant recruitment, interventions, and assessments is presented in **Figure 2**.

### Recruitment and informed consent

From January 2025 to January 2026, patients meeting the inclusion criteria will be recruited and registered at the Department of Breast Surgery of Yunnan Provincial Hospital of Traditional Chinese Medicine and The First Affiliated Hospital of Kunming Medical University. Follow-up will continue until June 2027. After being informed about the study and its potential risks and benefits, patients will be required to provide written informed consent prior to randomization, followed by eligibility screening.

### Participants

#### Inclusion criteria

1. Patients meeting the aforementioned diagnostic criteria with clinical stage II or III breast cancer;
2. Patients with invasive breast cancer and axillary lymph node metastasis confirmed by preoperative biopsy, who are scheduled to undergo axillary lymph node dissection (ALND);
3. Absence of preoperative upper limb lymphedema;
4. Female patients aged 18 to 75 years;
5. Life expectancy ≥ 3 years as clinically estimated by a physician;
6. Capable of self-care in daily life and able to cooperate to complete all interventions;
7. Participation is completely voluntary; patients must sign the informed consent form and be willing to cooperate with follow-up during the study period.

#### Exclusion criteria

1. Upper limb edema caused by other diseases, such as edema of cardiac, renal, or nutritional origin.
2. Patients currently using medications or undergoing other treatment regimens that conflict with this protocol.
3. Patients with coagulation disorders, severe mental disorders, or severe cardiovascular, cerebrovascular, hepatic, or renal diseases.
4. Physical disability of the affected limb, or a history of prior surgery or severe trauma on the affected limb.
5. Women who are pregnant or lactating.

### Removal criteria

Cases that were mistakenly included and do not meet the inclusion criteria.

1. Patients who receive other treatments during the study period (e.g., surgical treatments for breast cancer-related lymphedema, including lymphaticovenular anastomosis [LVA], debulking surgery, vascularized lymph node transfer [VLNT], or liposuction).
2. Patients with poor compliance or an insufficient observation period.
3. Patients with incomplete study data.
4. Patients who are lost to follow-up.
5. Patients who experience severe adverse events during the study period, or are diagnosed with other major diseases, tumor recurrence, or metastasis, rendering the study protocol no longer applicable.

### Sample size calculation

Given the novel and exploratory nature of the intervention for BCRL in this study, a minimum clinical sample size of 30 participants per group was selected (18),Assuming equal sample sizes for both groups and anticipating a 20% dropout rate, the total sample size was calculated as follows: N = 30o2×(1 + 20%)=72. Therefore, a total of 72 participants will be enrolled, with 36 participants in each group.(Reference: Whitehead AL, Julious SA, Cooper CL, Campbell MJ. Estimating the sample size for a pilot randomized trial to minimize the overall trial sample size for the external pilot and main trial for a continuous outcome variable. Statistical Methods in Medical Research. 2016;25 (3):1057-73).

### Randomization and blinding

Eligible patients will be randomly assigned in a 1:1 ratio to receive either TEAS or Sham TEAS treatment. An independent staff member will assist in establishing and managing the randomization database. Patients in both the TEAS and Sham TEAS groups will be blinded to their group allocation. During each session, they will receive treatment in a closed unit. Except for the acupuncturists, all other researchers, including statisticians, outcome assessors, and data analysts, will be blinded to the group allocation. Furthermore, the acupuncturists will not be involved in outcome assessment or data analysis.

### Intervention

Patients in both groups will receive basic treatment. Subsequently, they will undergo TEAS or Sham TEAS in separate treatment rooms to ensure no communication occurs between them. All participants will be informed that they may not perceive any sensation of stimulation during the procedure. All interventions will be administered by professional acupuncturists with at least 3 years of clinical experience who have completed standardized operational training. Both the observation and control groups will receive treatment once daily from the day before surgery until the third postoperative day (with treatment suspended on the day of surgery). Thereafter, treatments will be administered twice weekly for 4 weeks, totaling 12 intervention sessions.

### TEAS group

The treatment group will receive TEAS treatment.

Acupoint Selection: Tianfu (LU3), Taiyuan (LU9), Qingling (HT2), Shaohai (HT3), Xinshu (BL15), and Geshu (BL17) on the affected side. The localization of acupoints follows the Standard International Acupuncture Nomenclature proposed by the World Health Organization (WHO) (90/8579-Atar-8000) (19). Specific locations are listed in **Table 1**.

**Table 1:** Specific locations of acupoints.

Acupoints	Location of acupoints
Tianfu(LU3)	In the anterior region of the arm, radial border of the biceps, 3 cun below the head of the anterior axillary stripe
Taiyuan(LU9)	In the anterior region of the wrist, on the radial side of the transverse stripe on the distal palmar side of the wrist, at the fluctuation of the radial artery
Qingling(HT2)	In the anterior region of the arm, 3 inches above the transverse line of the elbow, in the medial measuring groove of the biceps
Shaohai(HT3)	In the anterior region of the elbow, with the elbow flexed, at the midpoint of the line between the medial end of the transverse elbow stripe and the medial epicondyle of the humerus
Xinshu(BL15)	In the spinal region, below the spinous process of the 5th thoracic vertebra, 1.5 cun from the posterior median line
Geshu(BL17)	In the spinal region, below the spinous process of the 7th thoracic vertebra, 1.5 cun from the posterior median line

Body Position: Patients will be placed in the lateral decubitus position for all selected acupoints: LU3, LU9, HT2, HT3, BL15, and BL17.

Procedure: A KD-2A Transcutaneous Electrical Nerve Stimulation Therapeutic Apparatus (Beijing Yaoyang Kangda Medical Instrument Co. Ltd.) will be used. The skin around the acupoints will be disinfected. After the skin is dry, positive and negative electrode pads will be applied to the acupoints respectively. LU3 and LU9 will form one pair; HT2 and HT3 will form a second pair; and BL15 and BL17 will form a third pair.

Treatment Parameters: The frequency is set to 2/100 Hz (dense-disperse wave) with a pulse width of 50dt The output intensity will be gradually increased to the patient’s maximum tolerated level. The treatment duration is 30 minutes per session.

Treatment Course: Preventive intervention begins on the first day before surgery and continues daily until the third day after surgery. Subsequently, all patients will receive scheduled treatments twice a week (every Monday and Thursday at 10:00 AM) for 30 minutes per session. This phase consists of 8 consecutive scheduled sessions, extending to one month post-operation, for a total of 12 interventions.

### Sham TEAS group

The control group will receive Sham TEAS treatment. The acupoint selection, connection of electrode pads, retention time, and duration of the treatment course are identical to those of the treatment group. However, the stimulation intensity for the control group is first adjusted to the patient’s lowest sensory threshold and maintained for 30 seconds. Subsequently, the intensity is gradually decreased over the next 15 seconds until there is no current output. This brief period of stimulation ensures the maintenance of blinding. Similar to the treatment group, the device’s indicator light will flash, and the buzzer will sound.

### Basic treatment

All patients will receive standard preventive measures for lymphedema following axillary lymph node dissection for breast cancer, including preventive Intermittent Pneumatic Compression (IPC) therapy and preventive Manual Lymphatic Drainage (MLD). Patients will be instructed on how to perform limb rehabilitation exercises and advised to observe specific precautions for the affected upper limb postoperatively. These precautions include avoiding blood pressure measurements, blood draws, and injections on the affected side; preventing burns, cuts, infections, and insect bites on the affected limb; avoiding saunas or hot baths; avoiding strenuous repetitive movements with resistance using the affected limb; and avoiding excessive fatigue or maintaining a fixed posture for prolonged periods. Additionally, nutritional support will be strengthened.

### Follow-up

Since the onset of lymphedema typically occurs between 3 months and 3 years postoperatively, peaking at 18 months (5), the follow-up period is established as 1.5 years postoperatively. From the completion of the intervention until 1.5 years postoperatively, follow-up will be conducted once a month via telephone or WeChat. The content of these follow-ups includes the visual analogue scale (VAS) for perceived swelling and lymphedema-related symptoms. Additionally, an in-person appointment will be scheduled at 1.5 years postoperatively to collect data on upper limb circumference, edema stage, and severity.

### Baseline characteristics of the participants

Sociodemographic Data: Name, gender, age, height, weight, education level, marital status, occupation, etc.

Disease-related Data: Tumor location, date of surgery, preoperative diagnosis, TNM staging, clinical stage, planned surgical method, whether preoperative neoadjuvant chemotherapy was performed, number of lymph nodes dissected, whether axillary radiotherapy is required during the intervention period, etc.

### Outcome measurement

#### Measurement timepoint

T0 (Baseline phase): Before treatment.
T1-T4 (Inpatient treatment phase): From the preoperative day to postoperative day 3.
T5-T12 (Outpatient treatment phase): From the 1st to the 8th scheduled visit.
T13-T29 (Follow-up phase): From the 1st to the 17th follow-up.

### Primary outcome

The incidence and time of onset of lymphedema from the postoperative period until the end of follow-up.

### Secondary outcomes

**Figure 3:**
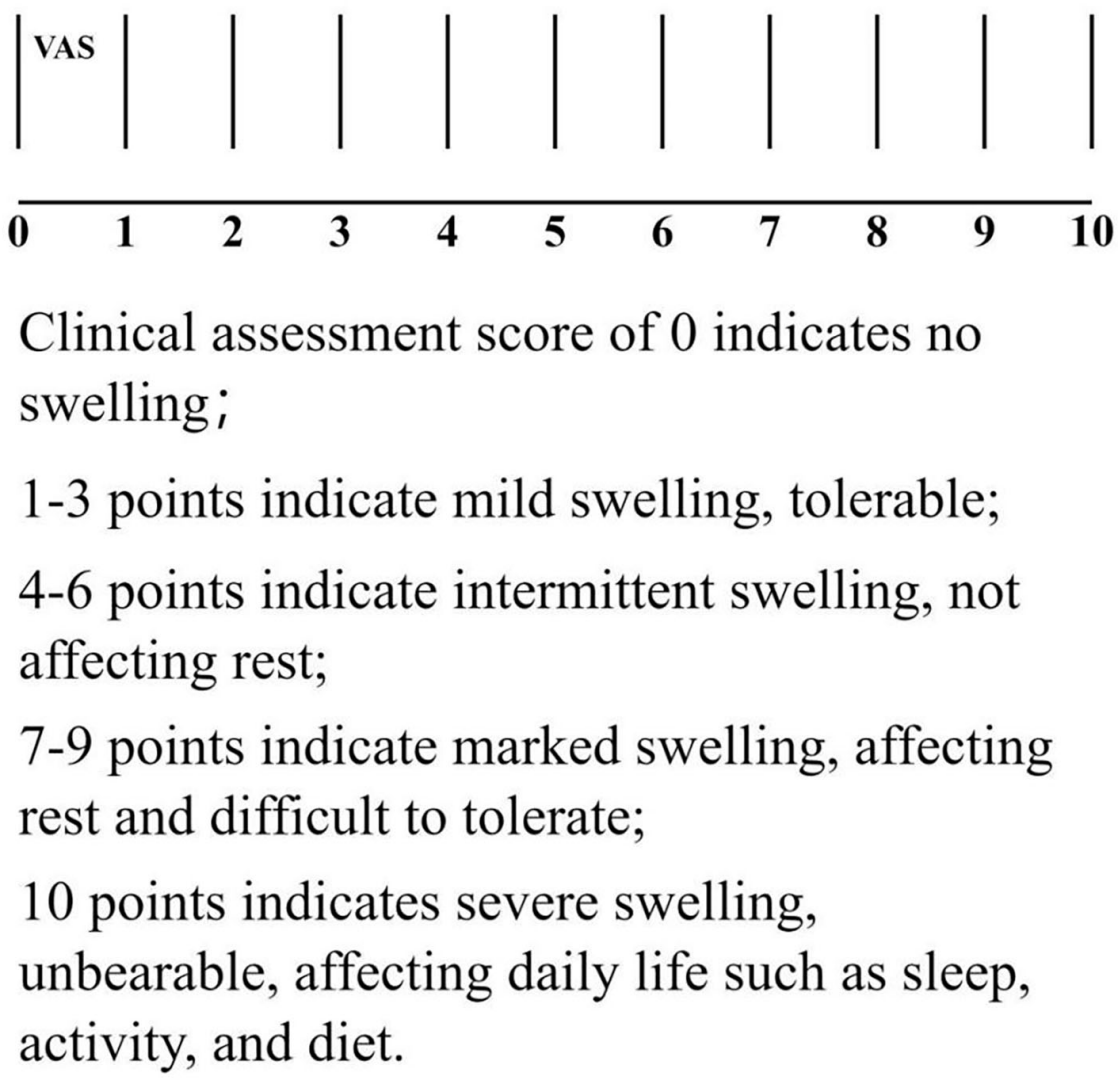
Visual simulation score.

Visual Analogue Scale (VAS) for Perceived Swelling: Changes in the VAS score for perceived swelling will be observed at the following time points: before treatment (T0), from preoperative to postoperative day 3 (T1-T4), upon completion of twice-weekly post-discharge treatments (T5-T12), and during monthly follow-ups until 18 months postoperatively (T13-T29). Data will be recorded in the VAS data collection form (see **Figure 3** and **Table 2**).

**Table 2:** Data collection form for visual analogue scale (VAS) of perceived swelling.

Name: Age: Operated limb: Date of surgery:					
Assessment timepoint	0 Points No swelling	1-3 Points mild swelling, tolerable	4-6 Points intermittent swelling, does not affect rest	7-9 Points marked swelling, affects rest, difficult to tolerate	10 Points severe swelling, unbearable, affects daily life (sleep, activity, diet, etc.)
Preoperative (T1)					
Postoperative Day 1 (T2)					
Postoperative Day 2 (T3)					
Postoperative Day 3 (T4)					
Scheduled Treatment 1 (T5)					
Scheduled Treatment 2 (T6)					
Scheduled Treatment 3 (T7)					
Scheduled Treatment 4 (T8)					
Scheduled Treatment 5 (T9)					
Scheduled Treatment 6 (T10)					
Scheduled Treatment 7 (T11)					
Scheduled Treatment 8 (T12)					
Follow-up 1 (T13)					
Follow-up 2 (T14)					
Follow-up 3 (T15)					
Follow-up 4 (T16)					
Follow-up 5 (T17)					
Follow-up 6 (T18)					
Follow-up 7 (T19)					
Follow-up 8 (T20)					
Follow-up 9 (T21)					
Follow-up 10 (T22)					
Follow-up 11 (T23)					
Follow-up 12 (T24)					
Follow-up 13 (T25)					
Follow-up 14 (T26)					
Follow-up 15 (T27)					
Follow-up 16 (T28)					
Follow-up 17 (T29)					

Lymphedema-related Symptoms: Lymphedema-related symptoms will be observed at baseline (T0), from preoperative to postoperative day 3 (T1-T4), upon completion of twice-weekly post-discharge treatments (T5-T12), and during monthly follow-ups until 18 months postoperatively (T13-T29). The symptom scale includes items such as swelling, tightness, heat, firmness, numbness, heaviness, tingling, pain, general discomfort, distress, and weakness. Each item is scored on a scale of 0 to 10, where 0 indicates “no sensation” and 10 indicates “intense sensation” (see **Table 3**).

**Table 3:** Data collection form for lymphedema-related symptom scores.

Name: Age: Operated limb: Date of surgery:											
Items	No sensation 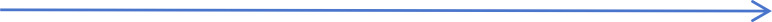								Intense sensation		
	0	1	2	3	4	5	6	7	8	9	10
Swelling											
Tightness											
Heat											
Firmness											
Numbness											
Heaviness											
Tingling											
Pain											
General discomfort											
Distress											
Weakness											
Total score											
Assessment timepoint	Swelling	Tightness	Heat	Firmness	Numbness	Heaviness	Tingling	Pain	General discomfort	Distress	Weakness
Preoperative (T1)											
Postoperative Day 1 (T2)											
Postoperative Day 2 (T3)											
Postoperative Day 3 (T4)											
Scheduled Treatment 1 (T5)											
Scheduled Treatment 2 (T6)											
Scheduled Treatment 3 (T7)											
Scheduled Treatment 4 (T8)											
Scheduled Treatment 5 (T9)											
Scheduled Treatment 6 (T10)											
Scheduled Treatment 7 (T11)											
Scheduled Treatment 8 (T12)											
Follow-up 1 (T13)											
Follow-up 2 (T14)											
Follow-up 3 (T15)											
Follow-up 4 (T16)											
Follow-up 5 (T17)											
Follow-up 6 (T18)											
Follow-up 7 (T19)											
Follow-up 8 (T20)											
Follow-up 9 (T21)											
Follow-up 10 (T22)											
Follow-up 11 (T23)											
Follow-up 12 (T24)											
Follow-up 13 (T25)											
Follow-up 14 (T26)											
Follow-up 15 (T27)											
Follow-up 16 (T28)											
Follow-up 17 (T29)											

Comparison of Limb Circumference Changes, Edema Staging, and Severity: Changes in the circumference, edema stage, and severity of the affected limb will be observed at baseline (T0), before and after treatments from preoperative to postoperative day 3 (T1a/b-T4a/b), before and after twice-weekly post-discharge treatments (T5a/b-T12a/b), and at 18 months postoperatively (T29). The mean values and differences in circumference before and after treatment will be compared between the two groups to evaluate the effect of treatment on limb edema. Staging and measurement standards are detailed in **Table 4**.

**Table 4:** Data collection form for upper limb circumference, edema staging, and severity.

Name: Age: Operated limb: Date of surgery:													
Upper limb circumference measurement							Edema stage				Edema severity		
Measurement timepoint	Measured limb	Palmar crease	Wrist crease	10 cm above wrist crease	Elbow crease	5 cm above elbow crease	Stage 0	Stage I	Stage II	Stage III	Mild	Moderate	Severe
Preoperative (T1)	L												
	R												
Postoperative Day 1 Pre-treatment T2a	L												
	R												
Postoperative Day 1 Post-treatment T2b	L												
	R												
Postoperative Day 2 Pre-treatment T3a	L												
	R												
Postoperative Day 2 Post-treatment T3b	L												
	R												
Postoperative Day 3 Pre-treatment T4a	L												
	R												
Postoperative Day 3 Post-treatment T4b	L												
	R												
Scheduled Treatment 1 Pre-treatment T5a	L												
	R												
Scheduled Treatment 1 Post-treatment T5b	L												
	R												
Scheduled Treatment 2 Pre-treatment T6a	L												
	R												
Scheduled Treatment 2 Post-treatment T6b	L												
	R												
Scheduled Treatment 3 Pre-treatment T7a	L												
	R												
Scheduled Treatment 3 Post-treatment T7b	L												
	R												
Scheduled Treatment 4 Pre-treatment T8a	L												
	R												
Scheduled Treatment 4 Post-treatment T8b	L												
	R												
Scheduled Treatment 5 Pre-treatment T9a	L												
	R												
Scheduled Treatment 5 Post-treatment T9b	L												
	R												
Scheduled Treatment 6 Pre-treatment T10a	L												
	R												
Scheduled Treatment 6 Post-treatment T10b	L												
	R												
Scheduled Treatment 7 Pre-treatment T11a	L												
	R												
Scheduled Treatment 7 Post-treatment T11b	L												
	R												
Scheduled Treatment 8 Pre-treatment T12a	L												
	R												
Scheduled Treatment 8 Post-treatment T12b	L												
	R												
18 Months Postoperative (T29)	L												
	R												

Edema Staging:.

ISL Staging classifies lymphedema into 4 stages based on severity:

Stage 0: Latent or subclinical stage; no obvious limb swelling, no visible clinical symptoms.

Stage I: Obvious swelling with pitting edema; swelling subsides upon limb elevation.

Stage II: Swelling does not subside with elevation; early stage presents as pitting edema, while late stage becomes non-pitting due to subcutaneous fat deposition and fibrosis.

Stage III: Non-pitting edema with elephantiasis; skin thickening, roughness, folds, hyperpigmentation, loss of elasticity, and severe fibrosis.

Edema Severity:

Generally determined by comparing the circumference of the affected limb to the healthy limb at the same measurement point:

Mild edema: Circumference difference < 3 cm

Moderate edema: Circumference difference 3–5 cm

Severe edema: Circumference difference > 5 cm

Side of Affected Limb:

L, Left

R, Right

Measurement Instructions:

The patient should be in a sitting or standing position with arms hanging naturally. Use a soft, non-elastic tape measure to encircle and measure the fixed points on both upper limbs. Measurement Points: Palmar crease, Wrist crease, 10 cm above wrist crease, Elbow crease, and 5 cm above elbow crease. Note: To avoid error, measure each point 3 times and calculate the average. Ensure measurements are taken at a fixed time, using the same tape measure, in the same position, and preferably by the same person to guarantee data accuracy and consistency.

Skin Thickness Ultrasound Examination: Changes in the skin thickness of the affected limb will be measured using ultra-high-frequency ultrasound. The specific measurement locations are detailed in **Table 5**. These assessments will be performed at baseline (T0), on postoperative day 1 (T2), after the 12th treatment (T12), and at 18 months postoperatively (T29).

**Table 5:** Patient upper limb skin thickness record form.

Name: Age: Operated limb: Date of surgery:						
Measurement of skin thickness in the patient’s upper limbs						
Assessment timepoint	Measured limb	Measurement point 1	Measurement point 2	Measurement point 3	Measurement point 4	Measurement point 5
Baseline (T0)	L					
	R					
Postoperative Day 1 (T2)	L					
	R					
Scheduled Treatment 8 (T12)	L					
	R					
18 Months Postoperative (T29)	L					
	R					

Side of Affected Limb:.

L, Left

R, Right

Measurement points:

(a) Arm positioned in supinated position: 1. Medial upper arm. 2. Lateral upper arm measuring points are placed 7 cm above the cubital crease. 3. Medial forearm. 4. Lateral forearm measuring points are placed 7 cm below the cubital crease. (b) Arm in pronated position: 5. Mid-point dorsal hand surface measuring point is placed between the wrist and the first metacarpophalangeal joint.

Measurement Instructions:

In the initial measurement position, participants were seated with their upper arm slightly abducted, elbow fully extended, and hand in supination. For the fifth measurement, participants placed their hands in a pronated position on the dorsal side of the hand.

Lymphangiography of the Affected Limb: Lymphatic system imaging will be performed at baseline (T0), on postoperative day 1 (T2), after the 12th treatment (T12), and at 18 months postoperatively (T29).

Serum Inflammatory Factor Levels: Peripheral venous blood samples will be collected at baseline (T0), on postoperative day 3 (T4), and at the end of treatment (T12). Serum levels of inflammatory factors, including Tumor Necrosis Factor-alpha (TNF-r-a high-sensitivity C-reactive protein (hs-CRP), and Procalcitonin (PCT), will be measured using ELISA. All blood samples will be sent to the laboratory for analysis and subsequently disposed of in accordance with relevant regulations.

### Safety evaluation

Monitoring and Recording of Adverse Events: Investigators will continuously monitor patient safety using specific questionnaires and record all adverse events (AEs) throughout the study process. Records will detail the relationship between the adverse events and the study treatment, onset and resolution dates, severity, actions taken, and whether the adverse event resulted in withdrawal from the trial. These findings will be summarized at the conclusion of the study.

Laboratory and Physical Examinations: Routine blood tests, liver function, renal function, and electrocardiography (ECG) will be performed at baseline and after 12 weeks of treatment.

### Data analysis

Software and Data Handling: All statistical analyses will be performed by an independent statistician using SPSS 25.0 software (IBM, Armonk, NY). Missing data will be imputed using the Last Observation Carried Forward (LOCF) method.

Normality and Continuous Variables: Measurement data will be assessed for normality using the Shapiro-Wilk (S-K) test. Data conforming to a normal distribution will be expressed as mean ± standard deviation (SD). Intra-group comparisons (pre- and post-treatment) will be analyzed using the paired samples t-test, while inter-group comparisons will be performed using the independent samples t-test. Measurement data that does not follow a normal distribution will be analyzed using non-parametric rank-sum tests.

Categorical Variables and Significance: Enumeration data (categorical data) will be presented as percentages or constituent ratios, and differences between groups will be analyzed using the Chi-square (χ²) test. All statistical tests will be two-sided, with the significance level set at αt 0.05. A P-value < 0.05 will be considered statistically significant.

### Data collection and management

All data will be initially recorded in Case Report Forms (CRFs) and subsequently entered into the study’s data management system by the researchers. Relevant patient characteristics, such as names, ID numbers, and telephone numbers, will be recorded in an anonymized format. For participants who discontinue treatment or deviate from the protocol, researchers will make every effort to supplement missing outcome data during the follow-up phase. The database will be locked after the last participant has completed the final follow-up and the data has been verified by two personnel; once locked, no modifications can be made. All participant files will be encrypted, stored, and centrally managed by the research center with strict confidentiality to ensure data security and traceability.

### Quality control

The design of this trial has been evaluated and refined by experts in acupuncture, breast surgery, statistics, and methodology. Any modifications to the trial protocol will be reported to the ethics committee, and any changes to the database will be recorded in the Case Report Forms (CRFs). Furthermore, to ensure trial quality, all researchers, including acupuncturists, data collectors, and statisticians, will undergo unified training. Licensed acupuncturists possessing a master’s degree in medicine and clinical experience are required to strictly adhere to standardized intervention procedures. All physicians must complete a one-day professional training session to familiarize themselves with the treatment protocols. To ensure consistency across participants, a set of clinical Standard Operating Procedures (SOPs) will be formulated. The aforementioned measures are intended to ensure the feasibility and safety of the clinical study.

### Trial status

This trial is currently in the recruitment phase. As of July 2025, 10 participants have been enrolled.

### Patient and public involvement

Patients and/or the public were not involved in the design, conduct, reporting, or dissemination plans of this research.

## Discussion

Breast cancer patients frequently undergo ALND due to the common occurrence of ipsilateral lymph node metastasis. However, this procedure disrupts lymphatic drainage channels, causing obstruction and the retention of protein-rich lymph in interstitial spaces, which increases fluid accumulation and ultimately induces BCRL (20). Although axillary lymph node dissection (ALND) is a critical factor in the development of breast cancer-related lymphedema (BCRL), its pathogenesis is not merely a consequence of mechanical obstruction; rather, it is a complex pathological process characterized by the decompensation of the lymphatic transport system following injury. Clinical observations reveal that only approximately one-quarter of patients develop lymphedema (3). This variability depends primarily on the individual’s lymphatic functional reserve and the efficiency of postoperative compensatory mechanisms. Research demonstrates that despite the anatomical disruption of lymphatic pathways caused by surgical resection, functional compensation can be achieved through enhanced contractility of residual lymphatic vessels, the opening of pre-existing anastomoses, or the establishment of collateral circulation (21). Only when the extent of surgical injury exceeds an individualnn compensatory limit, accompanied by pathophysiological changes such as interstitial inflammation, fat accumulation, and fibrosistion,gical in a lymphatic load that persistently exceeds the residual transport capacityttly, early functional impairment ultimately progress to clinically overt lymphedema (7). Furthermore, risk factors including obesity, radiotherapy, and the extent of lymph node dissection significantly compromise this compensatory capacity (6), further driving the heterogeneity of clinical incidence. Currently, there are no specific pharmacological treatments for BCRL; management primarily relies on physical therapies (e.g., manual drainage, compression bandaging) or surgical interventions. However, surgical treatments are costly, invasive, and difficult to implement widely, highlighting the urgent need for a safe and effective novel therapy (22). Research indicates that TEAS may alleviate edema and promote limb function recovery by improving lymphatic return, enhancing local blood circulation, and relieving pain and discomfort (17). Nonetheless, there is a lack of rigorous RCTs evaluating the efficacy of TEAS for BCRL, particularly those involving sham-TEAS controls. Therefore, this study aims to compare the efficacy and safety of TEAS versus sham TEAS in preventing BCRL, improving symptoms, and enhancing quality of life through a multicenter, double-blind RCT.

According to TCM theory, BCRL is not directly caused by the cancer itself but by damage to local lymphatic vessels and meridians during ALND. The Lung Meridian of Hand-Taiyin “emerges transversely from the lung system to the axilla,” the Heart Meridian of Hand-Shaoyin “emerges from the axilla,” and the Pericardium Meridian of Hand-Jueyin “descends to the axilla,” indicating that the axilla is the convergence point of the Three Yin Meridians of Hand. ALND primarily damages these meridians, leading to obstructed Qi flow in the axilla and upper limb. In this study, selected acupoints include Tianfu (LU3) and Taiyuan (LU9) of the Lung Meridian, Qingling (HT2), Shaohai (HT3), and Xinshu (BL15) of the Heart Meridian, and Geshu (BL17), which is the Influential Point of Blood, capable of resolving stasis and unblocking collaterals. The combination of these acupoints aims to improve lymphatic drainage and enhance local blood circulation.

The primary objective of this study is to determine the efficacy of TEAS for BCRL. In addition to recording subjective indices (e.g., VAS scores for perceived swelling and lymphedema-related symptoms) and objective clinical parameters (e.g., limb circumference changes and edema staging), this study highlights the crucial role of lymphatic system visualization and morphological monitoring in early diagnosis and assessment. Given that the initial pathological changes in lymphedema often manifest as slight thickening of the skin rather than an increase in subcutaneous tissue volume, this study incorporates ultrasound monitoring of skin thickness to provide early morphological evidence that is more sensitive than circumference measurements (23). Furthermore, to objectively assess the immediate impact of surgery on the lymphatic system, this study emphasizes the importance of preoperative baseline (T0) assessment. Serial near-infrared (NIR) fluorescence imaging, performed pre- and post-operatively (24), enables the dynamic observation of functional lymphatic vessel damage and the subsequent formation of collateral circulation. NIR possesses excellent tissue penetration, sensitivity, and high resolution, enabling non-invasive real-time tissue imaging. Indocyanine green (ICG) is a common NIR fluorescent dye used in BCRL assessment. Upon subcutaneous injection, ICG binds to plasma proteins in lymph, emitting detectable fluorescent signals that are transduced into lymphatic system images (25). The advantages of fluorescence imaging include the absence of ionizing radiation, minimal trauma, repeatability, real-time imaging, and ease of operation, making it suitable for preoperative assessment, intraoperative tracing, and postoperative follow-up. Furthermore, to comprehensively evaluate the intervention, this study will investigate the impact of TEAS on serum inflammatory factor levels. Finally, we will evaluate the statistical differences between TEAS and sham TEAS regarding BCRL incidence and quality of life.

This study has several limitations. Firstly, BCRL occurrence is closely related to tumor site, invasion extent, surgical method, and history of radiotherapy or chemotherapy; these confounding factors may influence result interpretation. Therefore, identifying independent risk factors for BCRL is crucial. Secondly, BCRL is not inevitable, and its onset varies from immediately post-surgery to months or years later; thus, universal preventive intervention for all patients may increase health economic costs. Furthermore, patient treatment compliance poses a challenge. Finally, regarding the placebo control design, although maintaining the minimum sensation threshold for 30 seconds helps achieve blinding, it may underestimate the short-term effects of TEAS. Conversely, reducing the current intensity increases the risk of unblinding. Future research should further optimize trial design, explore the combined efficacy of TEAS with other therapies, and evaluate its generalizability and long-term effects in diverse populations.
